# Supplementary material for: Socioeconomic inequalities in maternal healthcare utilization: An analysis of the interaction between wealth status and education, a population-based surveys in Tanzania
Source: PLOS Glob Public Health. 2023 Jun 13;3(6):e0002006. doi: 10.1371/journal.pgph.0002006 (PMC10263315; doi:10.1371/journal.pgph.0002006)
Supplement: Attachment — Submitted filename: Response to reviewers.docx [file pgph.0002006.s001.docx]

**Response to reviewers**

Reviewer #1:

**Comment 1**

Abstract
In the background section of abstract authors have mentioned that there is still huge inequalities in accessing maternal health service throughout pregnancy and postpartum periods. However, I this statement suggested as in this analysis the authors have measured the equity gap in utilization of service rather than access of service so suggest highlighting disparity in utilization rather than access in background section to make it more aligned with study purpose.

**Response:** Thank you for taking your time to review our manuscript and highlighting important issues for making this paper to publishable. The sentence has been revised and amended as suggested by the reviewers, [see page 2, Line 1 – 2].

**Comments 2**
Similarly, in the method section of abstract, there is a need to describe method a bit more in the method section of abstract specially describe which data has been analyzed (DHS) as part of data collection method, describe characteristic of sample population, size including Period of study as this analysis has taken 3 set of TDHS.
**Response:** Thank you for the comments, the Methods section in the abstract has been revised and amended as suggested by the reviewer, [see Page 2, Line 6 – 9].

**Comment 3**
Overall, the secondary analysis was conducted correctly with interesting findings. However, some of the findings can be a basis for further studies. For instance, according to Multivariate logistic regression models with an interaction term between wealth index and education there is no association between CSD and wealth and education however if we look at Descriptive statistics of women included in the analysis by maternal healthcare utilization indicators the rate of CSD in higher among the rich and richest quintile and among women with the secondary and higher-level education attainment. In general, CSD is higher in educated and wealthier as they often reach-out to the private sector also delay marriage, advance maternal age as educated often prefer delay marriage and pregnancy due to career related opportunity. Study from SSA also suggest https://www.ncbi.nlm.nih.gov/pmc/articles/PMC8975863/. CS and its association with educational attainment and wealth index however, this is not in the case of this analysis which need to be highlight in the discussion section and suggest for further study.

**Response:** Thank you for the careful observation regarding association between CSD and wealth status and education. In this study we discussed the interaction effect of wealth index and education by using adjusted predicted probabilities which showed clearer interpretation than multivariate analysis. However, you concern have been added as one of the limitations of this study, [see Page 21, Line 20 – 22 and Page 22, Line 1 – 4]

**Reviewer #2:**

**Comment 1**

Overall manuscript looks well written and is of importance to add knowledge on the coorelation of maternal healthcare with socioeconomic status and education of women, I have suggested some minor changes and I think this manuscript needs to be checked by a statistician to evaluate the findings of the study and validity of the methods used,

**Response:** Thank you for taking your time to review our manuscript and suggesting for minor changes. We tried for our best level to make all correction suggested by reviewers.
